# Milk composition changes and alterations in bacteria, serum, and gut metabolome over time in lactating yaks and Simmental cows

**DOI:** 10.5713/ab.25.0109

**Published:** 2025-06-10

**Authors:** Runze Wang, Yunxiang Xu, Allan Degen, Xuefeng Han, Xinsheng Zhao, Qunying Zhang, Yayu Huang, Binqiang Bai, Yingkui Yang, Shujie Liu, Yanfeng Xue, Lizhuang Hao

**Affiliations:** 1 Qinghai University, Key Laboratory of Plateau Grazing Animal Nutrition and Feed Science of Qinghai Province, Xining, China; 2 Desert Animal Adaptations and Husbandry, Wyler Department of Dryland Agriculture, Blaustein Institutes for Desert Research, Ben-Gurion University of the Negev, Beer Sheva, Israel; 3 Key Laboratory for Agro-Ecological Processes in Subtropical Region, National Engineering Laboratory for Pollution Control and Waste Utilization in Livestock and Poultry Production, South-Central Experimental Station of Animal Nutrition and Feed Science in Ministry of Agriculture, Hunan Provincial Engineering Research Center for Healthy Livestock and Poultry Production, Institute of Subtropical Agriculture, The Chinese Academy of Sciences (CAS), Changsha, China; 4 PEGASE, INRAE, Institut Agro, Saint-Gilles, France; 5 College of Animal Science and Technology, Anhui Agricultural University, Hefei, China

**Keywords:** Conjugated Linoleic Acid, Gut Microbiota, Milk Composition, Multi-omics

## Abstract

**Objective:**

This study aimed to elucidate the mechanisms underlying milk composition divergence between naks (female yaks) and Simmental cows (S-cows) by integrating longitudinal multi-omics analyses of gut microbiota and metabolomes.

**Methods:**

We determined the gut microbiota and metabolites of both species over a 54-day period (day 26 to 80 of lactation) of ten naks and ten S-cows. Gut microbiota dynamics were assessed via 16S rRNA sequencing, while serum and fecal metabolomes were profiled using ultra-high performance liquid chromatography–tandem mass spectrometry. Statistical analyses included Wilcoxon rank-sum tests, linear discriminant analysis effect size (linear discriminant analysis>2, p<0.05), and Spearman correlations (r>0.70).

**Results:**

Milk yield was lesser (0.53–0.91 vs. 2.07–3.88 kg/d) but concentrations of fat (5.63%–6.30% vs. 3.30%–3.74%), protein (5.66%–6.30% vs. 3.39%–3.74%), and conjugated linoleic acid (CLA) (1.74%–2.35% vs. 1.40%–1.75%) were greater (p<0.001) in nak than S-cow milk. Species-specific microbial signatures emerged. In naks, the *g-Family-XIII-AD3011-group* and *g-norank-Ruminococcaceae* were correlated with bile acid metabolism and CLA synthesis via 13-hydroxyoctadecadienoic acid transport. Additionally, the naks gut had a greater concentration of 13-hydroxyoctadecadienoic acid, a precursor of CLA, which may be transported to mammary cells via phosphatidylcholine and converted to CLA under the catalysis of fatty acid desaturase2. S-cows harbored *g-Succinivibrio* and *g-Eubacterium-ruminantium-group*, which are linked to galactose utilization and mTOR-mediated amino acid allocation. Metabolomics revealed naks-enriched steroid biosynthesis and taurine pathways (false discovery rate<0.05), while S-cows exhibited a lactating network associated with greater milk yield.

**Conclusion:**

Host-specific gut microbiota mediated nutrient allocation trade-offs. Naks optimized lipid-rich milk through bile acid and CLA metabolic networks, whereas S-cows enhanced yield via microbial-galactose synergies. This research underscores the pivotal role of the gut microbiome in mediating milk composition and suggests that microbiome manipulation could be a promising strategy to enhance milk quality in ruminants.

## INTRODUCTION

The yak (*Poephagus grunniens*), a ruminant native to the Qinghai-Tibetan Plateau (QTP), has evolved unique adaptations to thrive in extreme high-altitude environments characterized by low temperatures and hypoxia [1], and provides meat, milk, dung, and hides to local communities. Nak (female yak) milk, renowned for its high nutritional density, is described as a “concentrated milk” due to its elevated fat and protein content [2]. In contrast, the Simmental cow (S-cow), a dual-purpose breed originating from lowland regions and introduced widely to the QTP for production [3], is recognized for its superior meat quality and high milk yield, with an average annual production of approximately 3,000 kg [4,5]. S-cows have a greater milk yield than naks, but nak milk contains greater concentration of fat, protein, and conjugated linoleic acid (CLA) than S-cow milk. This divergence is reasoned to arise, at least in part, from species-specific gut microbiota modulating nutrient metabolism.

Gastrointestinal tract microbiota play an essential role in the digestion and absorption of nutrients, immune modulation, and overall health of ruminants, being closely linked with the host’s metabolic activities [6]. For instance, studies have reported the influence of gut microbiota on diarrhea in Holstein and S-cows [7], as well as the response of naks gut microbiota to environmental factors affecting diarrhea [8]. Additionally, research has indicated that the gut microbiota in naks influence the production of short-chain fatty acids (SCFAs), thereby affecting the flavor of nak milk [9]. However, the specific mechanisms by which gut microbiota govern milk composition warrants further studies, as prior research has relied predominantly on cross-sectional comparisons or single-omics approaches. Longitudinal multi-omics analyses linking microbial dynamics to milk metabolite profiles are lacking, particularly in high-altitude-adapted species.

To examine the mechanisms underlying milk quality divergence between naks and S-cows, this study integrated metabolomics and 16S rRNA gene sequencing to systematically compare their gut microbiota and metabolites during early lactation (days 26–80 postpartum). We hypothesized that species-specific gut microbiota drive milk composition differences, with nak-enriched microbial taxa promoting fat and protein synthesis, while S-cow microbiota prioritize yield-related pathways. Furthermore, lactation-associated shifts in microbial communities and metabolites are proposed to influence milk composition in a host-specific manner. These findings aim to provide novel insights into improving milk quality in dairy ruminants through targeted microbial interventions.

## MATERIALS AND METHODS

### Housing and feeding systems

The study was conducted from June to September, 2023, at the Huimu Yak Breeding Base in Qinghai Province, Tongpu Town, Wulan County, at an altitude of 3,300 m above sea level. Ten healthy early-lactating naks (142±8.3 kg), and ten healthy early-lactating S-cows (286±10.7 kg), approximately 4 years old, with an average parity of 2 calvings, were housed in the same barn, with each animal tethered in an individual pen. On non-sampling days, the calves and cows were kept together from 08:00 to 20:00 and the calves could suck, and were separated from 20:00 to 08:00 the following day. The females were hand milked at 08:00 for human consumption. The calves were also fed using an automatic milk feeder (Lely Calm; Lely).

The study consisted of a 20-day adaptation period followed by a measurement period of 54 days (days 26 to 80 of lactation). Throughout the study, both the naks and S-cows were provided with a ration formulated for lactating cows in accordance with the 2004 Chinese Beef Cattle Nutrient Requirements (Table 1). The animals were fed twice daily (between 6:00–7:00 and 17:00–18:00), with *ad libitum* access to drinking water throughout the day. The amount of feed offered was adjusted twice a week based on the feed intake, ensuring that 5% of the feed was left as refusal before the next feeding. Average daily intake was approximately 6 kg for naks and 13 kg for S-cows. Dietary composition and nutrient levels are shown in Table 1 (Qinghai Huanong Hengqing Agriculture and Animal Husbandry). The barn was disinfected each week and the animals were dewormed every half year with a broad-spectrum dewormer.

### Collection of milk, gastrointestinal tract and blood samples

Samples were collected on d 1, 24 and 54 (d 26, 50 and 80 of lactation) following the 20 d adjustment period. Prior to morning feeding, approximately 20 mL of jugular vein blood were collected in evacuated tubes, centrifuged at 4,000×g for 20 minutes, and the serum was stored in sterile centrifuge tubes at −20°C.

On sampling days, sampling methods for naks and S-cows were the same. The calves and cows were separated from 20:00 till 20:00 the following day. The females were hand milked by two-person teams for 5 min at 20:00 after the calves were allowed to suck freely and were then milked at 08:00, 12:00 and 20:00 the following day. The calves initiated milk letdown and were then removed at each of the three milkings, and the total was considered the daily (24 h) milk yield. The daily milk production was pooled, and 60 mL were stored in four 15 mL cryotubes at −20°C, and 20 mL were stored in ten 2 mL cryotubes at −80°C.

During the interval between milk sample collections, a fecal sample was collected manually from the rectum of each female and stored immediately in liquid nitrogen for later analysis.

### Metabolites in the gastrointestinal tract and serum

The fecal samples were processed according to Liu et al [10]. Approximately 50 mg of feces were placed in a pre-cooled grinding ball mill with liquid nitrogen and ground in pre-cooled methanol (CNW Technologies). The samples were incubated at 0°C for 10 minutes and at 220°C for 1 hour. After centrifugation at 13,000×g for 15 minutes at 4°C, 20 mL of supernatant was collected, mixed with 200 mL quality control sample, and injected into the liquid chromatography–tandem mass spectrometry (LC-MS/MS) system. LC-MS/MS analysis used an ACQUITY BEH Amide column (2.1 mm×100 mm, 1.7 μm; Waters) coupled with a Vanquish UHPLC system (Thermo Fisher Scientific) and a Q Exactive HF-X quadrupole-Orbitrap mass spectrometer (Thermo Fisher Scientific) [11].

Serum processing followed Wang et al [12]. Four hundred μL of 80% methanol were added to 100 μL of serum in an Eppendorf tube and shaken on ice for 5 minutes. The sample was centrifuged at 15,000×g for 5 minutes at 4°C, and the supernatant was collected, diluted to 53% methanol, and further diluted with mass spectrometry-grade water. The sample was centrifuged again at 15,000×g for 10 minutes at 4°C, and the supernatant was collected for LC-MS analysis. The ultra-high performance liquid chromatography–tandem mass spectrometry (UHPLC-MS/MS) analysis used a Vanquish UHPLC system (Thermo Fisher Scientific) coupled with a Q Exactive mass spectrometer (Thermo Fisher Scientific). A chromatographic column (100×2.1 mm, 1.9 μm) was used for the analysis of both fecal and serum samples, with a linear gradient elution over 17 minutes at a flow rate of 0.2 mL/min. The specific elution process was as follows: in positive ion mode, the mobile phase consisted of solvent A (0.1% formic acid in water) and solvent B (methanol). Gradient elution began with a high proportion of solvent A, gradually increasing the proportion of solvent B to achieve separation of the analytes. Specifically, the gradient started with 95% solvent A and 5% solvent B, ramping linearly over 15 minutes to 5% solvent A and 95% solvent B. The system then returned to the initial conditions for re-equilibration. In negative ion mode, solvent A was 5 mmol/L ammonium acetate, and solvent B was methanol. The solvent gradient was programmed as follows: 2% B for 1.5 min; 2%–100% B over 12.0 min; 100% B for 14.0 min; 100%–2% B from 14.1 to 17.0 min. The Q Exactive mass spectrometer was operated in both positive and negative ion modes, with a spray voltage of 3.2 kV, a capillary temperature of 320°C, a sheath gas flow rate of 40 arb, and an auxiliary gas flow rate of 10 arb.

For metabolite identification, raw data files generated from the UHPLC-MS/MS analyses were processed using Compound Discoverer 3.1 (CD3.1; Thermo Fisher Scientific) for peak extraction, alignment, and quantification of individual metabolites. The following parameters were set for the analysis: retention time tolerance of 0.2 min; mass accuracy tolerance of 5 ppm; signal intensity tolerance of 30%; signal-to-noise ratio of 3; and, minimum intensity threshold of 100,000. Peak intensities were subsequently normalized to the total ion intensity, and the results were matched against databases including mzVault, MassList, and mzCloud ( https://www.mzcloud.org/) for accurate identification and quantification. Further metabolite identification was confirmed using the KEGG and HMDB databases ( https://www.genome.jp/kegg/).

### DNA extraction, 16S rRNA gene amplification, and sequencing

DNA from gut content samples was extracted using the cetyltrimethylammonium bromide method. The bacterial 16S rRNA gene hypervariable regions (V3–V4) were amplified by universal bacterial primers 314F (5′-CCTAYGGGRBGCAS CAG-3′) and 806R (5′-GGACTACNNGGGTATCTAAT-3′). PCR products were detected by 2% agarose gel electrophoresis, and the target bands were excised and purified using a general-purpose DNA purification kit (TianGen). Library preparation used the NEB Next Ultra II FS DNA PCR-free Library Prep Kit (New England Biolabs), and the constructed libraries were quantified using both Qubit and Q-PCR. Upon verification of quality, paired-end sequencing (PE250) was conducted on a NovaSeq6000 platform. After sequencing, paired-end reads were merged, and barcode and primer sequences were trimmed using FLASH (v1.2.11) [13] with stringent parameters to ensure accurate overlap alignment. The processed data were then filtered using FLASp software (v.0.23.1) [13]. The resulting tags were aligned against a species annotation database to identify and remove chimeric sequences, ultimately obtaining the effective tags [14]. The effective data sequences were denoised using the DADA2 module with QIIME2 software (v.202006) by removing sequences with abundances less than 5, and thereby, generating amplicon sequence variants (ASVs) and the corresponding feature table. Data from each sample were subsequently normalized, using the sample with the smallest data quantity as the reference for normalization. Following sequence processing, the QIIME2 software package analyzed the ASVs further. Denoising was performed again with DADA2, and sequences with an abundance below 5 were removed. ASVs were then subjected to diversity analyses, including calculation of richness and alpha diversity indices, such as Chao1, Pielou’s evenness (Pielou-e), Shannon, and Simpson. UniFrac distances were calculated using QIIME2. To identify significant differences in species composition between groups, the linear discriminant analysis effect size (LEfSe) software was applied with a threshold for the linear discriminant analysis (LDA) score set at 2 and a significance level of p<0.05.

### Statistical analyses

The data were analyzed by R environment (v3.6.2), First, the metagenomeSeq package (v1.28.2) was used for cumulative sum scaling normalization of the raw data to correct for sequencing depth differences and, then, the microbiome package (v1.10.0) filtered out low-prevalence features (with a prevalence threshold of >10%). For the differential analysis of taxonomic units at the phylum, genus, and species levels, the Wilcoxon rank-sum test was applied in conjunction with the Benjamini-Hochberg false discovery rate (FDR) correction [15]. The LEfSe compared microbial abundance differences between naks and S-cows, with an LDA score>2 and p-value<0.05 considered statistically significant. Additionally, a t-test tested for metabolite differences between the two species, and metabolites with an FDR-corrected p-value<0.05 and variable importance in the projection (VIP)>1 were deemed significantly different.

In the metabolomics data analysis, Pareto-scaled LC-MS data underwent orthogonal partial least squares discriminant analysis (OPLS-DA) using the ropls package (v1.24.0). VIP scores were calculated via 7-fold cross-validation. Differential metabolites were ultimately identified based on the criteria of FDR-corrected p-value<0.05 and VIP>1.

## RESULTS

### Milk yield and composition of naks and Simmental cows

Milk production was greater (p<0.001) in S-cows than naks throughout the study. Fat and protein contents as a percentage of milk yield were greater (p<0.001) in nak milk than in S-cow milk. The CLA concentration in nak milk was higher (p = 0.02) than in S-cow milk on days 24 and 54 (Table 2).

### Gastrointestinal tract microbial composition in naks and Simmental cows

**Figure 1:**
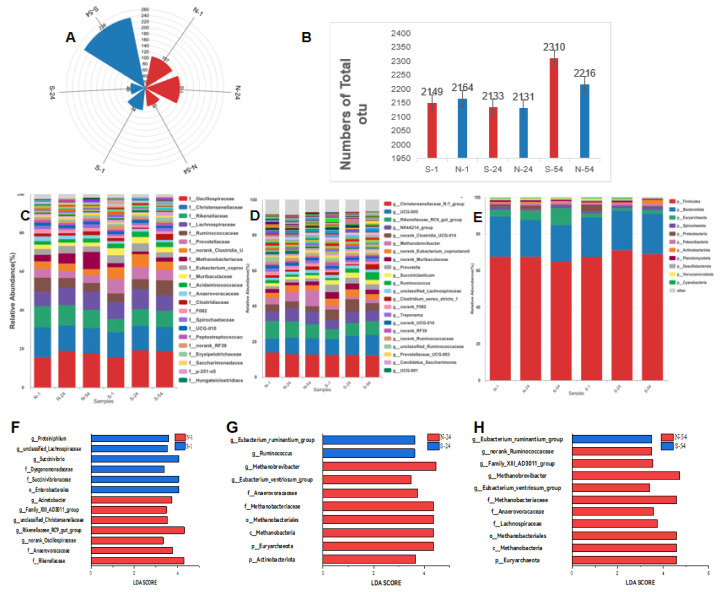
The gut microbiota composition in naks and Simmental cows on days 1, 24, and 54 of daily milk yield measurements (day 1 is day 26 of lactation). S represents Simmental cows, and N represents naks. (A) Venn diagrams illustrating the unique operational taxonomic units (OTUs) of Naks and Simmental cows. (B) Histograms of the OTUs of nak and Simmental cows. (C–E) Histograms of the gut microbiota composition of naks and Simmental cows at the phylum, genus, and species levels. (F–H) Linear discriminant analysis effect size (LEfSe) analysis of the gut microbiota of naks and Simmental cows.

The microbial communities were visualized using a Rose plot, which depicted unique operational taxonomic units (OTUs) for each species, as well as the total number of OTUs (Figures 1A, 1B). The abundance of unique gut microbiota in naks increased from day 1 to 24, surpassing the S-cows; whereas, the abundance of gut microbiota in S-cows decreased. By day 54, this trend reversed, and the microbiota of S-cows increased.

At the phylum level, in naks, the relative abundances of p-Firmicutes and p-Bacteroidota increased, while of p-Euryarchaeota decreased between days 1 and 54. In S-cows, the relative abundances of p-Firmicutes and p-Bacteroidota increased between days 1 and 24, and then decreased, whereas p-Euryarchaeota displayed an opposite trend (Figure 1E). At the family level, the abundances of *f-Oscillospiraceae* and *f-Christensenellaceae* exhibited similar trends in both naks and S-cows, increasing from day 1 to day 24, and then decreasing from day 24 to day 54. The relative abundance of *f-Rikenellaceae* decreased in naks from day 1 to 54, while in S-cows, the abundance increased and then decreased (Figure 1C).

At the genus level, the relative abundance of *g-ChristensenellaceaeR-7group* decreased from day 1 to day 54 in both naks and S-cows. In naks, the relative abundance of *g-UCG-005* increased initially and then decreased, while in S-cows, the relative abundance continued to increase. Furthermore, *g-Rikenellaceae-RC9-gut-group* decreased in naks but increased in S-cows over the same period (Figure 1D).

Based on LEfSe, on day 1, *g-Rikenellaceae-RC9-gut-group* was dominant in naks; whereas, *g-Succinivibrio* was dominant in S-cows (Figure 1F). On day 24, *g-Methanobrevibacter* was dominant in naks; whereas, *g-Ruminococcus* and *g-Eubacte-ruminantium-group* were dominant in S-cows (Figure 1G). On day 54, *g-Methanobrevibacter* remained dominant in naks, while *g-Eubacte-ruminantium-group* was dominant in S-cows.

### Serum and gastrointestinal tract metabolomics in naks and Simmental cows

We employed high-throughput LC-MS to determine the metabolomes in plasma and feces in naks and S-cows. A total of 948 serum metabolites and 1317 gut metabolites were identified. These metabolites included primarily fatty acids and lipids, amino acids and their derivatives, organic acids, carbohydrates, microbial nucleosides, and nucleotides.

**Figure 2:**
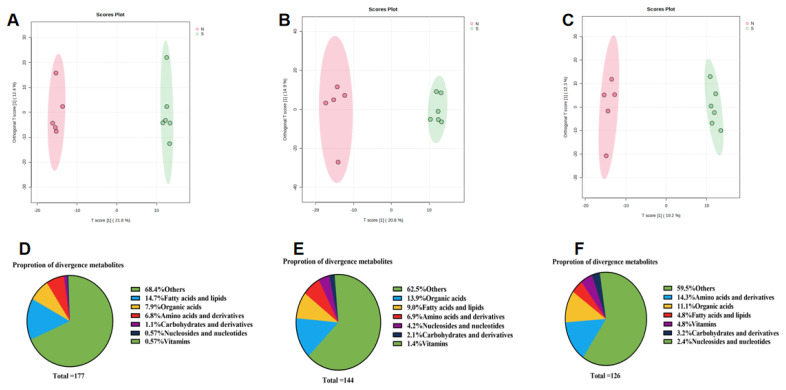
Differential metabolite profiles between naks and Simmental cows on days 1, 24, and 54 of daily milk yield measurements (day 1 is day 26 of lactation). S represents Simmental cows, and N represents Naks. (A–C) Orthogonal partial least squares discriminant analysis (OPLS-DA) of differential metabolites in the serum of naks and Simmental cows. (D–F) Pie chart of chemical properties of different metabolites in serum of naks and Simmental cows.

**Figure 3:**
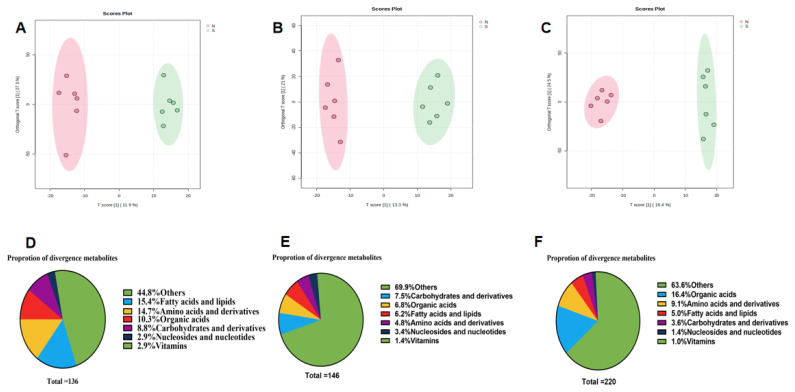
Gut differential metabolite map of naks and Simmental cows on days 1, 24, and 54 of daily milk yield measurements (day 1 is day 26 of lactation). S represents Simmental cows, and N represents naks. (A–C) Orthogonal partial least squares discriminant analysis (OPLS-DA) of differential metabolites in the gut of Naks and Simmental cows. (D–F) Pie chart of chemical properties of different metabolites in gut of naks and Simmental cows.

OPLS-DA revealed a clear separation between the fecal and plasma metabolomes of naks and S-cows. Specifically, for serum metabolites, the first and second principal components explained 21.8% and 12.4%, 20.8% and 14.9%, and 19.2% and 12.3% of the total variance for measurements taken on days 1, 24, and 54, respectively (Figures 2A–2C). For gut metabolites, the first and second principal components explained 11.9% and 27.3%, 13.3% and 21%, and 16.4% and 24.5% of the variance for days 1, 24, and 54, respectively (Figures 3A–3C). Validation of the OPLS-DA model exhibited no sign of overfitting, indicating that the model described sample variation accurately and could be used reliably for further data analysis.

By combining p-values, VIP scores, and fold changes (FC), we identified 177, 144, and 126 differentially abundant metabolites in the three sets of serum samples, respectively (p<0.05, VIP>1 and FC>2 or <0.5). When categorized by metabolite class, the differences in serum metabolites between naks and S-cows were driven primarily by changes in fatty acids, lipids, and amino acids. Specifically, in the day 1, 24, and 54 samples, fatty acids and lipids accounted for 39.5%, 34.7%, and 32.5% of the differential metabolites, while amino acids and their derivatives accounted for 6.8%, 6.9%, and 16.7%, respectively (Figures 2D–2F).

For the three sets of gut samples, we identified 136, 146, and 220 differentially abundant metabolites (p<0.05, VIP>1 and FC>2 or <0.5). Again, the primary differences in metabolite composition were observed in fatty acids, lipids, and amino acids. Fatty acids and lipids accounted for 15.4%, 6.2%, and 5.0% of the differential metabolites, while amino acids and their derivatives accounted for 14.7%, 4.8%, and 9.1%, respectively (Figures 3D–3F).

**Figure 4:**
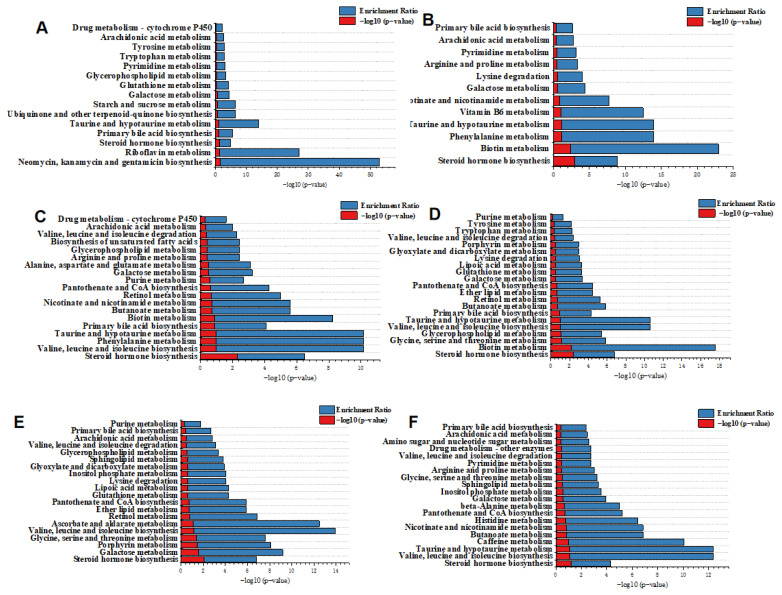
The KEGG pathway enrichment analysis diagram of naks and Simmental cows on days 1, 24, and 54 of daily milk yield measurements (day 1 is day 26 of lactation). (A–C) represent the KEGG pathway enrichment analysis diagram of serum differential metabolites, and (D–F) represent the KEGG pathway enrichment analysis diagram of gut differential metabolites.

To further investigate differences in serum metabolism between naks and S-cows, we used an enrichment analysis of differential serum metabolites (Figure 4). Using MetaboAnalyst 6.0, differential metabolic pathways were ranked according to the impact factor (impact) and p-value (Figure 4). On day 1 of measurements, the differential metabolites were enriched primarily in steroid biosynthesis, biotin metabolism, glycine, serine, and threonine metabolism, and taurine and hypotaurine metabolism (Figure 4A). On day 24 of measurements, the differential metabolites were concentrated mainly in steroid biosynthesis, galactose metabolism, porphyrin metabolism, and glycine, serine, and threonine metabolism (Figure 4B). By day 54 of measurements, the main metabolic pathways included steroid hormone biosynthesis, taurine and hypotaurine metabolism, and butyrate metabolism (Figure 4C). Notably, steroid hormone biosynthesis was enriched significantly across all measurements.

On day 1 of measurements, the differential gut metabolites were enriched primarily in neomycin, kanamycin, and gentamicin biosynthesis, and riboflavin, taurine and hypotaurine metabolism (Figure 4D). On day 24, the differential metabolites were involved mainly in biotin, phenylalanine, taurine, hypotaurine, and vitamin B6 metabolism (Figure 4E). On day 54, the metabolites were involved predominantly in the biosynthesis of valine, leucine, and isoleucine, and metabolism of phenylalanine, taurine and hypotaurine (Figure 4F). It is noteworthy that taurine and hypotaurine metabolism were enriched significantly in all measurements.

### Integrated analysis of microbiome, and serum and gastrointestinal metabolomes in naks and Simmental cows

To further identify potential interactions between metabolites and microorganisms and milk components, Spearman’s rank correlation tested relationships between gastrointestinal and serum metabolome and microbiome; 11 significant positive correlations emerged (r>0.70).

**Figure 5:**
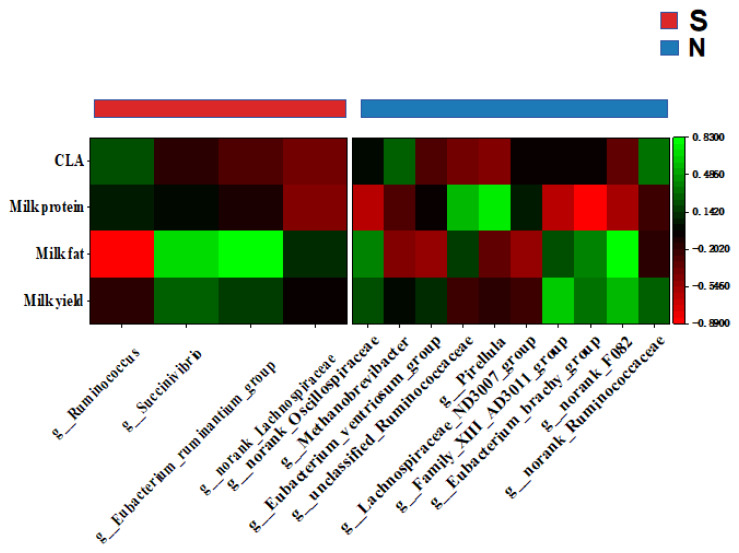
Spearman correlation coefficients between gut microbes and milk composition in naks (N) and Simmental cows (S) depicted by a correlation analysis heat map. Green to red blocks represent high to low correlations.

**Figure 6:**
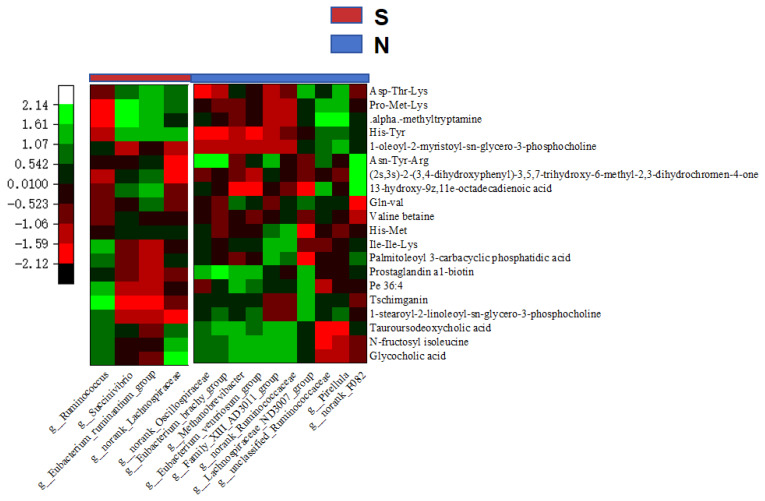
Spearman correlation analysis heat map between gut microbes and the top 20 abundant gut metabolites in naks (N) and Simmental cows (S). Green to red represent high to low correlations.

**Figure 7:**
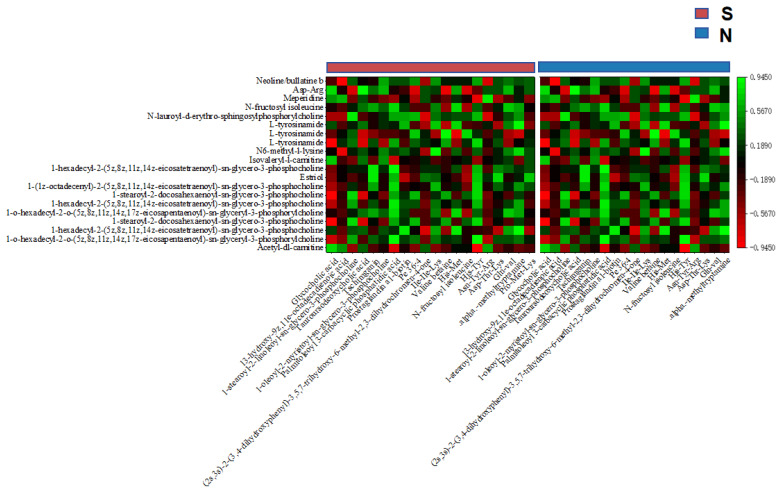
Heat map of Spearman correlation analysis between the top 20 abundant gut metabolites and the top 20 abundant serum metabolites in naks (N) and Simmental cows (S). Green to red represent high to low correlations.

**Figure 8:**
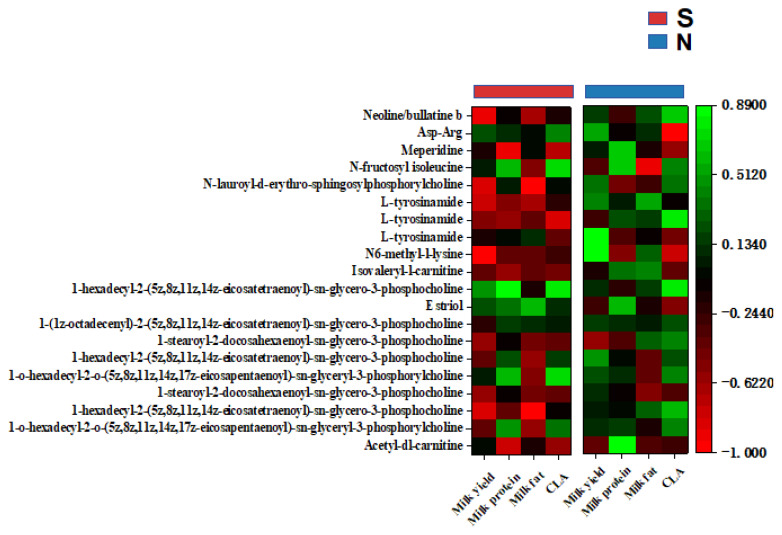
Heat map of Spearman correlation analysis between milk composition and the top 20 serum metabolites in abundance in naks (N) and Simmental cows (S). Green block to red block represent high to low correlation.

In S-cows, *g-Succinivibrio* was correlated positively (r> 0.70) with milk yield, fat, and CLA, and with the gut metabolites Asp-Thr-Lys and Pro-Met-Lys. *g-Eubacterium-ruminantium-group* was correlated positively with milk fat and the gut metabolites Pro-Met-Lys. *g-Ruminococcus* was correlated positively with milk CLA, and the gut metabolites His-Met and prostaglandin A1-biotin (Figures 5, 6). Furthermore, the gut metabolites Asp-Thr-Lys and Pro-Met-Lys were correlated positively with the serum metabolite estriol (r>0.70), while His-Met and Prostaglandin A1-biotin were correlated positively with the serum metabolite 1-hexadecyl-2-(5z,8z,11z,14z- eicosatetraenoyl)-sn-glycero-3-phosphocholine (Figure 7). The serum metabolite 1-hexadecyl-2-(5z,8z,11z,14z-eicosatetraenoyl)-sn-glycero-3-phosphocholine was correlated positively with milk protein, CLA, and yield (r>0.70). Additionally, the serum metabolites 1-o-hexadecyl-2-o-(5z,8z, 11z,14z,17z-eicosapentaenoyl)-sn-glyceryl-3-phosphorylcholine and N-fructosyl isoleucine were correlated positively with CLA (r>0.70), while estriol was correlated positively with milk fat (Figure 8).

In naks, positive correlations emerged between *g-Family-XIII-AD3011-group* and milk yield (r>0.70), between *g-norank-F082* and milk fat, and between *g-Pirellula* and milk protein. Both *g-norank-Ruminococcaceae* and *g-Methanobrevibacter* were correlated positively with CLA (Figure 5). *g-Family-XIII-AD3011-group* and *g-norank-Ruminococcaceae* were correlated positively with the gut metabolite glycocholic acid (r>0.70), while *g-norank-F082* was correlated positively with the gut metabolites 13-hydroxy-9z,11e-octadecadienoic acid, Palmitoleoyl 3-carbacyclic phosphatidic acid, (2s,3s)-2-(3,4-dihydroxyphenyl)-3,5,7-trihydroxy-6-methyl-2,3-dihydrochromen-4-one, and Asn-Tyr-Arg. *g-Methanobrevibacter* was correlated positively with the gut metabolite N-fructosyl isoleucine (Figure 6). The gut metabolites glycocholic acid and palmitoleoyl 3-carbacyclic phosphatidic acid were correlated positively with the serum metabolites 1-hexadecyl-2-(5z,8z,11z,14z-eicosatetraenoyl)-sn-glycero-3-phosphocholine and N-lauroyl-d-erythro-sphingosylphosphorylcholine (r>0.70). N-fructosyl isoleucine was correlated positively with the serum metabolite N-lauroyl-d-erythro-sphingosylphosphorylcholine (r>0.70), while 13-hydroxy-9z,11e-octadecadienoic acid was correlated positively with the serum metabolite Isovaleryl-l-carnitine. In addition, (2s,3s)-2-(3,4-dihydroxyphenyl)-3,5,7-trihydroxy-6-methyl-2,3-dihydrochromen-4-one was correlated positively with the serum metabolite neoline/bullatine b. The serum metabolites N6-methyl-l-lysine and Asp-Arg were correlated positively with the gut metabolite Asn-Tyr-Arg (Figure 7) (r>0.70). Serum metabolites 1-hexadecyl-2-(5z,8z,11z,14z-eicosatetraenoyl)-sn-glycero-3-phosphocholine and N-lauroyl-d-erythro-sphingosylphosphorylcholine were correlated positively with milk yield, milk fat, and CLA, while Isovaleryl-l-carnitine was correlated positively with milk fat and milk protein. Neoline/bullatine b was correlated positively with CLA (r>0.70). Furthermore, serum metabolites Asp-Arg and N6-methyl-l-lysine were correlated positively with milk yield (Figure 8) (r>0.70).

## DISCUSSION

We hypothesized that differences in microbiome composition, function, and metabolomics between naks and S-cows would result in differences in their milk yield and composition. To test this hypothesis, we determined the microbiome composition, function, and metabolomics of naks and S-cows, and their effects on milk yield and composition. The milk yield was greater in S-cows than in naks, but the concentrations of fat, protein, and CLA were greater in naks than S-cows. Despite the relatively low milk yield in naks, their fat content fell within the averages reported for different nak breeds [16,17]. Bacteria were consistently the most dominant gut microorganisms in both ruminant species, reinforcing the hypothesis that microbiota plays a critical role in milk fat and protein synthesis, consistent with earlier findings [18].

Through a longitudinal comparison of gut microbiota and serum, intestinal metabolites between naks and S-cows, we identified temporal changes in the relative abundances of bacteria phyla. From day 1 to 54 of measurements (approximately day 26 to 80 of lactation), the microbiome of naks exhibited a decrease in the relative abundances of Firmicutes and Bacteroidota, and an increase in Euryarchaeota, suggesting a shift in microbial communities. In contrast, S-cows displayed an initial increase in Firmicutes and Bacteroidota between days 1 and 24, followed by a decrease between days 24 and 54, while Euryarchaeota exhibited an opposite trend. These differences point to potential host-specific gut microbiome responses despite the same diet [19]. Both Firmicutes and Bacteroidota are involved in cellulose degradation and the production of volatile fatty acids, which indirectly influence milk fat synthesis, while Euryarchaeota is associated with nitrogen metabolism and influences milk protein synthesis [20]. However, although these phylum-level changes were observed, the trends in milk fat and protein concentrations were similar between naks and S-cows, suggesting that phylum-level analyses of gut microbiota may not provide sufficient insights into their effects on milk quality. However, genus-level analyses revealed intersting patterns. For instance, *g-Christensenellaceae-R-7-group* [21] and *g-Rikenellaceae-RC9-gut-group* [22], which were reported to correlate negatively with milk yield, exhibited distinct trends. The relative abundance of *g-Christensenellaceae-R-7-group* decreased steadily from day 1 to day 54, while *g-Rikenellaceae-RC9-gut-group* declined in naks but increased in S-cows before declining. These patterns were temporally aligned with milk yield, although the precise mechanisms remain unclear. As both genera are involved in preventing inflammation, we reasoned that they contribute to gut health and nutrient absorption, which indirectly enhances milk synthesis [23]. Notably, the increase in Firmicutes and Bacteroidota in S-cows was accompanied by an increase in *g-Olsenella*, a genus associated with improved gut health. While direct evidence linking this genus to reduced mastitis risk is limited, these findings suggest that *g-Olsenella* may play a role in reducing mastitis susceptibility in S-cows [24,25].

Based on serum metabolomics, lipid metabolites accounted for a greater proportion of differential metabolites between naks and S-cows. Specifically, naks had a greater concentration of serum fatty acid metabolites than S-cows, and this could explain the higher fat content in nak milk, as milk fat is synthesized primarily from serum non-esterified fatty acids [26]. Furthermore, differences emerged in the steroid hormone biosynthesis pathway between naks and S-cows. In serum, the concentration of estrone sulfate (E1S) was greater, while estradiol (E2) was lesser in naks than S-cows. E1S is derived from the conversion of estrone and estradiol [27], suggesting that the conversion rate of E2 to E1S might be faster in naks than in S-cows. Estradiol (E2), the primary estrogen secreted by the ovaries, stimulates mammary gland development and prepares the animal for lactation [28], which partly explains the greater milk yield in S-cows than naks.

Metabolisms of glycine, serine, threonine, taurine, and hypotaurine were enriched in the current study. L-proline betaine, a key metabolite in the glycine, serine, and threonine metabolic pathways, mediates protein synthesis via the mTOR pathway. The serum levels of L-proline betaine and taurine were greater in naks than S-cows, which may help explain the relatively greater protein content in naks than S-cow milk.

The taurine and hypotaurine metabolism pathways were enriched at all time points. Additionally, the fecal concentration of taurocholate in S-cows was greater than in naks. Taurine has been reported to influence mammary cell function and milk synthesis [29] and to have a positive effect on maintaining mammary gland health [30]. Our findings suggest that taurine may support mammary gland health in S-cows, thus contributing to their greater milk yield. At different time points, the concentration of phenylethylamine in the gut of naks increased gradually and was greater than in S-cows. High concentrations of biogenic amines have been linked to an increased likelihood of mastitis [31], which, in conjunction with the changes in the gut microbiota of *g-Christensenellaceae-R-7-group* and *g-Rikenellaceae-RC9-gut-group*, suggests that S-cows may have a stronger ability to maintain a healthy mammary gland than naks. Differences in the riboflavin metabolism pathway were observed between naks and S-cows. The greater riboflavin concentration in the gut of naks may contribute to their high milk fat content [32], but can also reduce milk yield [33], which is consistent with the findings in the present study. Gut biotin concentration exhibited a decreasing trend in the naks, with levels lesser than in the S-cows. A positive correlation between biotin and milk yield was reported [34], which is also consistent with the lesser milk yield in naks than S-cows.

Through a comparative analysis of the intestinal microbiota and metabolites between naks and S-cows, this study revealed that the divergence in milk quality stemmed primarily from differences in the composition of their gut microbial communities and the metabolic regulatory networks they drive. In the regulation of milk fat and CLA, the significant enrichment of *g-Family-XIII-AD3011-group* and *g-norank-Ruminococcaceae* in the nak gut suggests their potential role in optimizing lipid metabolism strategies to enhance milk fat content. Previous studies have indicated that such microbes may improve intestinal lipid absorption by promoting the conversion of bile acids to secondary bile acids [35], thereby enhancing bile acid metabolism efficiency [36]. Concurrently, the marked increase in the abundance of 13-hydroxyoctadecadienoic acid (a CLA precursor) in the nak gut suggests that this metabolite may be transported to mammary cells via phosphatidylcholine (1-hexadecyl-2-(5Z,8Z,11Z,14Z-eicosatetraenoyl)-sn-glycero-3-phosphocholine) and converted to CLA under the catalysis of fatty acid desaturase 2 (FADS2) [37,38], providing a partial molecular explanation for the high-fat characteristics of nak milk. In contrast, the enrichment of Succinivibrio in the S-cows gut elevateed the concentration of the Asp-Thr-Lys tripeptide. Although studies have confirmed that threonine (Thr) can promote the expression of fat synthesis-related genes by activating the mTOR pathway (via phosphorylation of downstream targets S6K1 and 4EBP1) [39,40], the direct regulatory mechanisms of tripeptides on the mTOR pathway require further investigation.

In terms of milk protein regulation, the high-protein characteristics of nak milk are closely associated with the enrichment of *g-norank-F082* and *g-Pirellula* in the nak gut. Specifically, the abundance of *g-norank-F082* correlated positively with the Asn-Tyr-Arg tripeptide in the gut, suggesting its potential role in supporting protein synthesis by providing essential amino acid precursors. Additionally, the elevated serum levels of isovaleryl-L-carnitine may enhance mitochondrial β-oxidation efficiency, thereby supplying energy for mammary protein synthesis [41]. Furthermore, *g-Pirellula* may indirectly promote milk protein synthesis by mediating ammonia nitrogen metabolism to improve nitrogen utilization efficiency [42]. In contrast, the enrichment of *g-Ruminococcus* in the S-cows gut generates SCFAs through fiber degradation [43], which may activate AMP-activated protein kinase (AMPK) in mammary epithelial cells [44], leading to the preferential utilization of amino acids for lactose synthesis and, consequently, reducing milk protein content.

The enrichment of *g-Succinivibrio* and *g-Eubacterium-ruminantium-group* in the S-cows gut may drive high milk production through dual patways. It can enhance galactose metabolism efficiency to optimize energy utilization [45], and, with the accumulation of tripeptides such as Asp-Thr-Lys and Pro-Met-Lys, it can activate the JAK2-STAT5 signaling pathway [46], which is closely associated with lactation regulation. However, this high-yield strategy may come at the metabolic cost of reduced nutritional density.

## CONCLUSION

In conclusion, the present study provides insights into the mechanisms underlying milk quality differences between naks and S-cows, which are primarily reflected in differences in their gut microbiota, the complexity of their metabolic pathways, and the dynamic regulatory networks between gut and serum metabolites. Naks optimize milk fat and CLA contents through more complex metabolic regulatory networks, particularly bile acid and fatty acid metabolic pathways, The significant enrichment of *g-Family-XIII-AD3011-group* and *g-norank-Ruminococcaceae* in the nak gut indicates their potential role in promoting the conversion of bile acids to secondary bile acids, thereby enhancing bile acid metabolism efficiency. The marked increase in the abundance of 13-hydroxyoctadecadienoic acid, a CLA precursor, in the nak gut indicates that this metabolite may be transported to mammary cells via phosphatidylcholine and converted to CLA under the catalysis of FADS2, providing a molecular explanation for the high-fat and high-CLA characteristics of nak milk. S-cows enhanced milk yield through specific microbial communities and metabolites. These findings provide important insights into improving dairy product quality and suggest potential targets and strategies for future nutritional interventions.

## Figures and Tables

**Table 1:** Composition and chemical composition of diets offered to the lactating females (dry matter [DM] basis except for DM content, which is on a fresh matter basis)

Feed components	Contents (% of DM)
Highland barley lees	60
Corn straw	20
Sea buckthorn seed meal	4
Corn	5
Rice bran	3
Corn germ meal	2
Corn steep liquor	3
Stone powder	0.6
NaHCO_3_	0.5
Urea	0.2
NaCl	0.2
Premix^1)^	1.5
Chemical composition	
Dry matter (% fresh matter)	60.2
Crude protein	14.2
Ether extract	3.76
Neutral detergent fiber	41.9
Acid detergent fiber	22.7
Ash	6.09
Calcium	0.93
Phosphorus	0.59
Gross energy (MJ/kg DM)	18.4

1) Premix contains vitamin A: 100 KIU; vitamin D3: 30 KIU; vitamin E: ≥300 IU; CuSO_4_: 275 mg; ZnSO_4_: 700 mg; FeSO_4_: 400 mg; and MnSO_4_: 500 mg/kg.

**Table 2:** Milk yield and milk composition of naks and Simmental cows collected on days 1, 24 and 54 (days 26, 50 and 80 of lactation) after the 20 day adaptation period

Day of measurement	Ruminant species	Milk yield (kg/d)	Protein content (%)	Fat content (%)	Conjugated linoleic acid (%)
1	Simmental	2.07	3.39	3.58	1.40
	Nak	0.53	5.63	6.30	1.74
	SEM	0.07	0.12	0.16	0.25
	p-value	<0.001	<0.001	<0.001	0.196
24	Simmental	3.88	3.58	3.44	1.69
	Nak	0.77	5.68	6.05	2.35
	SEM	0.08	0.15	0.21	0.24
	p-value	<0.001	<0.001	<0.001	0.02
54	Simmental	3.74	3.74	3.30	1.75
	Nak	0.91	5.82	5.66	2.27
	SEM	0.07	0.12	0.23	0.18
	p-value	<0.001	<0.001	<0.001	0.02
