# Corrigendum

**DOI:** 10.5713/ab.25.0109C

**Published:** 2026-02-24

A portion of the Materials and Methods section of **Anim Biosci Vol 38, No 11:2428–2442** was updated as follows.

**Previous Materials and Methods:**

Ten healthy early-lactating naks (142±8.3 kg), and ten healthy early-lactating S-cows (286±10.7 kg), approximately 4 years old, with an average parity of 2 calvings, were housed in the same barn, with each animal tethered in an individual pen.

**Revised Materials and Methods:**

Ten healthy early-lactating naks and ten healthy early-lactating Simmental cows (S-cows), approximately 4 years old, with an average parity of two calvings, were housed in the same barn, with each animal tethered in an individual pen. For subsequent measurements, six naks (140±5.6 kg) and six S-cows (332±17.3 kg) were randomly selected.

